# Post-Intensive Care Unit Multidisciplinary Approach in Patients with Severe Bilateral SARS-CoV-2 Pneumonia

**DOI:** 10.7150/ijms.77792

**Published:** 2023-01-01

**Authors:** Ana María Sánchez-García, Pilar Martínez-López, Adela María Gómez-González, Jorge Rodriguez-Capitán, Francisco-Javier Pavón-Morón, Rafael José Jiménez-López, José Manuel García-Almeida, Elma Avanesi-Molina, Nicolás Zamboschi, Carolina Rueda-Molina, Victoria Doncel-Abad, Ana Isabel Molina-Ramos, Eva Cabrera-César, Imad Ben-Abdellatif, Marina Gordillo-Resina, Esteban Pérez-Mesa, María Nieto-González, Pilar Nuevo-Ortega, Carmen Reina-Artacho, Pedro Luis Sánchez-Fernández, Manuel Francisco Jiménez-Navarro, María Antonia Estecha-Foncea

**Affiliations:** 1 Instituto de Investigación Biomedica de Malaga y Plataforma en Nanomedicina (IBIMA Plataforma BIONAND), Malaga, Spain; 2 Intensive Medicine Service, Hospital Universitario Virgen de la Victoria, Malaga, Spain; 3 Physical Medicine and Rehabilitation Service, Hospital Clínico Universitario Virgen de la Victoria, Malaga, Spain; 4 Centro de Investigacion Biomedica en Red de Enfermedades Cardiovasculares (CIBERCV), Instituto de Salud Carlos III, Madrid, Spain; 5 Cardiology and Cardiac Surgery Service, Hospital Universitario Virgen de la Victoria, Malaga, Spain; 6 Family and Community Medicine Service, Hospital Universitario Virgen de la Victoria, Malaga, Spain; 7 Cardiology Service, Hospital Universitario Virgen de la Victoria, Malaga, Spain; 8 Mental Health Service, Hospital Universitario Virgen de la Victoria, Malaga, Spain; 9 Pneumology Service, Hospital Universitario Virgen de la Victoria, Malaga, Spain; 10 Servicio de Cardiología, Hospital Universitario de Salamanca, Universidad de Salamanca, IBSAL, Salamanca, Spain

**Keywords:** COVID-19, respiratory failure, sequela, multidisciplinary

## Abstract

**Background:** Short and long-term sequelae after admission to the intensive care unit (ICU) for coronavirus disease 2019 (COVID-19) are to be expected, which makes multidisciplinary care key in the support of physical and cognitive recovery.

**Objective:** To describe, from a multidisciplinary perspective, the sequelae one month after hospital discharge among patients who required ICU admission for severe COVID-19 pneumonia.

**Design:** Prospective cohort study.

**Environment:** Multidisciplinary outpatient clinic.

**Population:** Patients with severe COVID-19 pneumonia, post- ICU admission.

**Methods:** A total of 104 patients completed the study in the multidisciplinary outpatient clinic. The tests performed included spirometry, measurement of respiratory muscle pressure, loss of body cell mass (BCM) and BCM index (BCMI), general joint and muscular mobility, the short physical performance battery (SPPB or Guralnik test), grip strength with hand dynamometer, the six-minute walk test (6-MWT), the functional assessment of chronic illness therapy-fatigue scale (FACIT-F), the European quality of life-5 dimensions (EQ-5D), the Barthel index and the Montreal cognitive assessment test (MoCA). While rehabilitation was not necessary for 23 patients, 38 patients attended group rehabilitation sessions and other 43 patients received home rehabilitation.

**Endpoints:** The main sequelae detected in patients were fatigue (75.96%), dyspnoea (64.42%) and oxygen therapy on discharge (37.5%). The MoCA showed a mean score compatible with mild cognitive decline. The main impairment of joint mobility was limited shoulder (11.54%) and shoulder girdle (2.88%) mobility; whereas for muscle mobility, lower limb limitations (16.35%) were the main dysfunction. Distal neuropathy was present in 23.08% of patients, most frequently located in lower limbs (15.38%). Finally, 50% of patients reported moderate limitation in the EQ-5D, with a mean score of 60.62 points (SD 20.15) in perceived quality of life.

**Conclusions:** Our findings support the need for a multidisciplinary and comprehensive evaluation of patients after ICU admission for COVID-19 because of the wide range of sequelae, which also mean that these patients need a long-term follow-up.

**Impact on clinical rehabilitation:** This study provides data supporting the key role of rehabilitation during the follow-up of severe patients, thus facilitating their reintegration in society and a suitable adaptation to daily living.

## Introduction

Coronavirus disease 2019 (COVID-19) is a relatively new disease in the public health context, which was first notified in Wuhan (China) in December 2019. The WHO declared COVID-19 a pandemic on 11 March 2020 because of its alarming levels of propagation and severity 1,2. The impact of this health emergency has increased the load on healthcare systems and the demand for rehabilitation during and after the disease 3.

On the other hand, the 2010 meeting of the Society of Critical Care Medicine coined the term "post intensive care syndrome" (PICS) to describe the physical, cognitive and mental health deficiencies that present after admission to intensive care once the acute phase of disease has passed 4. In addition, all survivors benefit from multidisciplinary management by intensive-care specialists, neuropsychiatrists, physiotherapists and respiratory therapists, who may employ both pharmacological and non-pharmacological measures 6,7.

Before the SARS-CoV2 pandemic, studies had described the sequelae after admission for adult respiratory distress syndrome, reporting a significant proportion of surviving patients with considerable sequelae in their functional capacity and state of health in their first months of recovery 8.

The arrival of the COVID-19 pandemic led to an exponential increase in the number of critical patients requiring intensive care. As a consequence, the intensive care units (ICUs) were overwhelmed by patients with severe hypoxia secondary to bilateral pneumonia and many of them had serious and multifactorial disabling sequelae. These sequelae were associated with a prolonged stay in ICU, respiratory distress 9, aggressive therapies (e.g., use of experimental treatments, turning patients to the prone position...) and direct effects of the coronavirus. To date, a dozen studies have evaluated different aspects of these sequelae, such as cognitive decline 10, motor effects that require rehabilitation and their impact on quality of daily life 11 (e.g., pain or discomfort, anxiety or depression, self-care and usual activity), perceived level of fatigue 12, intolerance to exercise, dyspnoea on moderate exertion and asthenia, in addition to limitations on physical capacity, respiratory function deterioration and its influence on psychological health 13,14. All of which make a multidisciplinary approach necessary for these patients because of the complexity of their symptoms. Above and beyond the final medical objective of recovering pulmonary function, there are other goals, such as the functional recovery and reintegration in and suitable adaptation to life in society 2,15,16.

This new reality has led some hospitals to create tools to facilitate the follow-up of these complex patients. In June 2020, the Hospital Universitario Virgen de la Victoria (Malaga, Spain) established a multidisciplinary circuit consisting of the main specialties involved in the treatment of COVID-19 and its medical consequences. During the first visit, one month after hospital discharge, sequelae were detected at a one-stop clinic where interventions were initiated. In the subsequent follow-up visits, both sequelae and interventions were monitored.

The aim of this study was to evaluate the sequelae found at one month after hospital discharge among patients with COVID-19 severe bilateral pneumonia from a multidisciplinary approach with different specialists. In the initial post-ICU visit, ICU specialists and, subsequently, specialists in physical medicine and rehabilitation, endocrinology and nutrition, mental health and pneumology.

## Methods

### Study design

An observational retrospective study of patients who required admission to the ICU of the Hospital Universitario Virgen de la Victoria (Malaga, Spain) between April 2020 and October 2021 for severe COVID-19 pneumonia.

### Participants

All of the patients admitted to the ICU had indications for aggressive measures, with a need for oxygen > 15 L/min and partial pressure of oxygen in arterial blood (PaO2) < 200 mmHg. Our study included survivor patients, who were given appointments for the post-COVID-19 multidisciplinary care circuit, where they were assessed at a "one-stop clinic" by specialists in physical medicine and rehabilitation, endocrinology, pneumology, mental health and intensive care. Because the Hospital Universitario Virgen de la Victoria is a referral centre for other health districts, we excluded both patients who had been referred from other hospitals as a consequence of pressure on the healthcare system within the epidemiological context and foreign patients because of the difficulty of completing their follow-up.

### Variables from the ICU

Using the medical records of the patients, we compiled the baseline sociodemographic characteristics, comorbidities, treatment required during their stay in the ICU, type of life support, mechanical ventilation, need for prone positioning, muscle relaxation, treatment received and complications after hospital admission. We also collected outcomes from the assessments of different specialists and the need for the inclusion of these patients in the rehabilitation programme, whether in group sessions or at home. The assignation of rehabilitation groups was based on the functional status of patients and the perceived sequelae at the first evaluation.

During a one-year follow-up period, we collected data from patients at the first and subsequent 3-, 6-, 9- and 12-month visits. Some patients did not require follow-up because they improved and were discharged from the rehabilitation programme. Not all patients required evaluation by all of the specialists included in the programme.

### Variables from the multidisciplinary care circuit

The clinical protocol of the multidisciplinary care included:

- Medical history with details of quality of life before COVID-19, independence for activities of daily living, need for assistance when walking, fatigue, physical exercise habits, pain and weakness.
- Data collected during hospitalisation: duration of hospitalisation (days), ICU stay (days), administered respiratory therapy, duration of mechanical ventilation (days), treatment given, duration of muscle relaxant exposure (days) and cycles of prone positioning.
- Data collection on sequelae:
  - Otorhinolaryngological sequelae.
  - Psychological/psychiatric sequelae: those sequelae that were described in the mental health visit using a clinical interview, and a record of the use of benzodiazepines and antidepressants consumed.
  - Respiratory sequelae: the need for oxygen therapy on discharge and at subsequent check-ups, dyspnoea (modified medical research council [mMRC] dyspnoea scale), spirometry (forced vital capacity [FVC], forced expiratory volume in first second of forced breath [FEV1] and FEV1/FVC ratio) 17 and respiratory muscle pressure (maximal inspiratory pressure [MIP] and maximal expiratory pressure [MEP]) 18. MIP mainly evaluates the force of inspiratory muscles (e.g., the diaphragm), whereas MEP evaluates the force of the muscles that contribute to expiration (e.g., the intercostal and abdominal muscles). Normal values for MIP were those greater than or equal to 75 cm H2O in men and 50 cm H2O in women, and normal values for MEP were those greater than or equal to 100 cm H2O in men and 80 in women.
  - Nutritional sequelae 19-21: Weight loss after hospital admission (kg) and changes in body cell mass (BCM) and BCM index (BCMI).
- Physical examination and tests:
  - Physical examination: General joint mobility and general muscular mobility.
  - The short physical performance battery (SPPB or Guralnik test) 22. The SPPB is an assessment tool for evaluation of lower extremity functioning and consists of three different tests: balance, walking speed and standing up, and sitting down in a chair five times. The score is between 0 and 12, and a score < 10 indicates frailty and high risk of disability and falls.
  - Grip strength with a hand dynamometer 23,24. The most commonly used and accepted method to assess grip strength; it is recognised by the American Society of Hand Therapists.
  - The six-minute walk test (6-MWT) 25. The 6-MWT assesses distance (walking less than 350 m in 6 minutes is associated with an increase in mortality among patients with chronic pulmonary diseases, heart failure or pulmonary hypertension), desaturation (prognostic indicator for interstitial lung disease) and number of stops.
- Quality of life questionnaires:
  - The functional assessment of chronic illness therapy-fatigue scale (FACIT-F) 26,27.
  - The European quality of life-5 dimensions questionnaire (EQ-5D) 19. The EQ-5D is a standardised instrument for health-related quality of life that is self-administered for mobility, self-care, usual activities, pain/discomfort, anxiety/depression. The visual analogue scale (VAS) is also used to assess perceived quality of life.
  - The Barthel index 28. This test measures the ability to perform basic activities of daily living and level of independence. It is a good predictor of mortality and response to rehabilitation treatments and assesses the functional outcomes and capacity to return to activities of daily life.
  - The Montreal cognitive assessment test (MoCA) 29,30. The MoCA was used for evaluation of sequelae from a cognitive perspective. This test evaluates eight cognitive domains: attention, concentration, working memory and short-term memory, visuospatial abilities, verbal fluency, language and executive functions. The maximum score is 30 and the proposed cut-off point 26, with a sensitivity of 90% and a specificity of 87%.

### Ethical considerations

The study was approved by the Regional Ethics Committee of the Hospital Universitario Virgen de la Victoria (project Id. POSTUCI21-1654-N-21) in accordance with the Ethical Principles for Medical Research Involving Human Subjects adopted in the Declaration of Helsinki by the World Medical Association (64th WMA General Assembly, Fortaleza, Brazil, October 2013) and the Regulation (EU) 2016/679 of the European Parliament and of the Council 27 April 2016 on the protection of natural persons with regard to the processing of personal data and on the free movement of such data (General Data Protection Regulation). Therefore, alphanumeric codes were used by the specialists and researchers during the evaluation sessions and preparation of database in order to maintain privacy and confidentiality of the participants. All patients who were included in the present study gave informed consent for the use of their anonymised data in the knowledge that this was a descriptive study for baseline characteristics. They also authorised their long-term follow-up. Patients were able to revoke their informed consent and withdraw from the study whenever they wished. Only one patient did not wish to participate in the study and did not sign the informed consent.

### Statistical analysis

The baseline sociodemographic and clinical characteristics of the patients are expressed as a mean and standard deviation [mean (SD)] for quantitative variables, and absolute values with percentages [n (%)] for qualitative variables. The sequelae and results derived from tests/questionnaires at the clinic during the follow-up were also expressed using quantitative (continuous or discrete) and qualitative variables. All statistical analyses of the database were performed using the IBM SPSS Statistics version 24 (IBM, Armonk, NY, USA).

## Results

### Participants

A total of 360 patients with severe COVID-19 pneumonia were admitted to the ICU of the Hospital Universitario Virgen de la Victoria from April 2020 to October 2021 and the multidisciplinary post-ICU clinic included 104 patients who completed the study. Of the remaining patients, 127 died during the hospital admission, 74 did not meet the inclusion criteria (33 patients were transferred from other hospitals, 40 patients were foreigners and one patient did not wish to participate in the follow-up) and 55 were lost to follow-up after discharge.

### Sociodemographic and clinical characteristics

We collected the baseline sociodemographic and clinical characteristics of the 104 patients, which are shown Table 1.

#### Sociodemographic data

The patients were predominantly male (77.9%) and the mean age was 56.7 years (SD 13.8). According to the usual body mass index (BMI) before the hospital admission, the patients were mainly classified into class 1 obesity (30.8%) and overweight (29.8%), followed by class 2 obesity (17.3%), class 3 morbid obesity (15.4%) and normal weight (6.7%). Regarding smoking habits, 44.2% of patients described themselves as former smokers and 3.8% were smokers.

#### Comorbidity and previous medical complications

The most prevalent comorbidity in these patients was arterial hypertension (48.1%), followed by dyslipidaemia (30.8%) and diabetes mellitus (27.9%). However, there were other previous medical complications that were also considered in the study. Thus, 23 patients (22.2%) had a previous history of respiratory problems: obstructive sleep apnoea (OSA) (10.6%), asthma (8.7%) and chronic obstructive pulmonary disease (COPD) (2.9%); 11 patients (10.6%) had a cardiological history: ischaemic heart disease (4.8%), arrhythmias (2.9%), valvular heart disease (1.9%) and hypertensive cardiomyopathy (1.0%); 8 patients (7.7%) had a history of orthopaedic problems: prior fractures (3.8%), osteoarthritis (1.9%) and myalgia (1.0%); and only 2 patients had a history of kidney disease (1.9%).

#### Therapeutic procedures for COVID-19

The mean stay in the ICU was 21.9 days (SD 19.6) and the mean period of hospitalisation was 47.3 days (SD 49.6). Regarding the clinical procedures of these patients, invasive mechanical ventilation was necessary for 53.8% of patients, and 46.2% of patients required high-flow nasal cannula therapy. All of the patients who required mechanical ventilation underwent an initial therapy with high-flow nasal cannula and the mean delay in orotracheal intubation from admission was 1.3 days (SD 2.2). The mean duration of mechanical ventilation was 27.1 days (SD 21.6) and tracheotomy with prolonged orotracheal intubation was necessary for 60.71% of patients who had mechanical ventilation.

The mean number of prone positioning cycles was 3.7 (SD 2.1).

The pharmacological treatment administered during the ICU stay was primarily tocilizumab (58.7%) and glucocorticoids (98.0%), followed by other drugs employed exclusively during the first wave of COVID-19, such as hydroxychloroquine (21.2%), lopinavir-ritonavir (19.2%) and interferon beta (17.3%). During the admission, 6.7% of patients received muscle relaxants for a mean period of 7.7 days (SD 6.7).

### Sequelae after hospital discharge

A multidisciplinary team assessed the sequelae after hospital discharge from different specialities (Table 2).

#### Nutritional sequelae

In nutritional terms, there was a weight loss of 10.8 kg (SD 7.9) and an active BCMI of 9.6 kg (SD 2.5) in men and 9.5 kg (SD 6.5) in women.

#### Otorhinolaryngological sequelae

Important otorhinolaryngological sequelae were observed in these patients (22.11%). Specifically, the patients were mainly diagnosed with aphonia (13.5%), followed by aural or glottic granuloma (2.9%), tracheal stenosis (2.9%), hearing loss (1.9%) and tracheostomy fistula (1.0%).

#### Psychological and psychiatric sequelae

Regarding the psychological/psychiatric abnormalities, we found important sequelae related to mood disorders and emotional responses. Thus, 17 patients (16.3%) were diagnosed with anxiety and/or depression during the follow-up: anxiety (5.8%), depression (2.9%) and mixed anxiety-depressive disorder (11.5%); and 12 patients showed adjustment disorder (11.5%). In contrast, 11 patients showed a condition compatible with a normal experiential reaction (10.6%). Secondary to these mood abnormalities, 26.0 % of patients reported insomnia and an elevated consumption of psychotropic drugs (28.8% benzodiazepines and 26.9% antidepressants).

#### Respiratory sequelae

From a respiratory perspective, we assessed mMRC dyspnoea and the need for home oxygen therapy on hospital discharge. There were different degrees of dyspnoea in 64.4% of patients: grade 1 in 31.7%, grade 2 in 24.0% and grade 3 in 8.7% of patients. On hospital discharge, 37.5% of patients required home oxygen therapy and 8.7% were still receiving this therapy at the six-month follow-up visit.

#### Sequelae related to joint and muscular mobility

Among the patients with impaired joint mobility (36.5%), the main limitation of joint mobility was observed at shoulder (11.5%) and shoulder girdle (2.9%) level. The examination of muscular mobility revealed sequelae in 51.9% of patients, and the main sequelae were lower limb (16.4%), upper limb (5.8%) and general (6.7%) limitations.

#### Sequelae of pain

Twenty-eight patients reported painful conditions (26.9%). Mainly, neuropathic pain (13.5%), meralgia paresthetica (7.7%), multiple enthesopathies (5.8%) and lumbar-sciatic pain (4.8%.). Distal neuropathy was present in 23.1% of patients, more frequently in lower limb (15.4%), and brachial plexopathy was only reported in patients who required mechanical ventilation (5.8% of total).

#### Functional sequelae

Regarding the physical state, 19 patients required aid to walk at the first visit (18.3%) using a walking stick or frame (15.4%) or wheelchair (2.9%). The degree of fatigue/tiredness reported by the patients was predominantly fatigue on moderate effort (48.1%) followed by fatigue on great (18.3%) and mild (9.6%) efforts. The Barthel index for activities of daily living showed a score of 91.50 points (SD 16.31), which indicates a moderate-slight dependency.

In terms of cognition, the MoCA test was conducted on 47 patients and the mean score was > 26 points, which is compatible with mild cognitive deterioration. A second analysis of the MoCA test after adjusting for age revealed worse results in patients with ≥ 65 years than in patients under 65 years with a mean score of 16 points (SD 9) and 22 points (SD 9), respectively.

### Rehabilitation and follow-up

The patients who underwent a one-year follow-up at the multidisciplinary post-ICU clinic were divided into three groups according to the rehabilitation procedures. Thus, 23 patients did not require rehabilitation (22.1%), 38 patients required group rehabilitation sessions (36.5%) and 43 patients received home rehabilitation (41.4%) with a set of exercises to follow.

#### Functional testing

As shown in Table 3, functional testing was conducted in the rehabilitation clinic and consisted of the following tests: the SPPB (Guralnik test), the 6-MWT and grip strength with a hand dynamometer. The SPPB score revealed that 27.1% of patients were frail individuals. In the 6-MWT, 27.0% of patients did not walk a normal distance (> 350 m), 13.0% of patients needed at least one stop and 6.5% required oxygen therapy during 6 minutes of walking. Regarding the hand dynamometer, the mean pressure force was 25.1 kg (SD 10.6) with the right hand and 22.4 kg (SD 11.3 kg) with the left hand.

#### Respiratory functional testing

The respiratory functional testing was performed with spirometry and measurements of MIP and MEP in order to evaluate the respiratory muscle strength (Table 4). The spirometry parameters of FVC and FEV1 were > 80% and the FEV1/FVC ratio was 75.1% (SD 12.2).

In addition to spirometry, measurements of MIP and MEP were conducted on the patients considering sex. While men had a mean MIP of 81.6 cm H2O (SD 29.8) and a mean MEP of 95.6 cm H2O (SD 41.6), women had a mean MIP of 68.8 cm H2O (SD 44.0) and a mean MEP of 70.2 cm H2O (SD 22.2). The MIP values were normal for both sexes but the MEP values were lower than the reference values (100 cm H2O for men and 80 cm H2O for women).

#### Quality of life

The EQ-5D showed a perceived quality of life of 60.6 points (SD 20.2) and high percentage of negative responses (i.e., "No problems") in the different dimensions of the questionnaire (Table 5); specifically, for mobility (62.5%), self-care (67.9%), usual activities (50.0%) and anxiety/depression (64.3%). For pain, 50.0% of patients reported moderate problems and 44.6% no problems.

Finally, a phone survey was conducted six months after hospital discharge to complete the FACIT-F scale and 53.8% of patients reported no fatigue, 24.0% mild fatigue, 13.5% intense fatigue and 7.7% moderate fatigue.

## Discussion

Prior to the COVID-19 pandemic, the post-intensive therapy syndrome or PICS had been defined as the deterioration or impairment in physical, cognitive and/or mental health that arises after a critical illness and persists beyond discharge from the ICU 6. Therefore, a comprehensive and integrated multidisciplinary follow-up is essential for patients who have been admitted to ICU 31. The rehabilitation after a severe disease or long hospitalisation is key to minimise the disabling effects of the associated sequelae in order to optimise the extent of independence and maximize the reintegration of the patients in society 32.

A wide range of publications have reported sequelae in survivors of severe acute respiratory distress syndrome (ARDS) regarding fatigue, impaired physical function, anxiety and depression 12. Thus, ARDS is associated with high morbidity and mortality in the acute phase, followed by major sequelae in the general functional capacity and health state in the first months of recovery 8,33,34. A study with a cohort of survivors of COVID-19 in a rehabilitation clinic reported clinically significant levels of psychological symptoms such as depression, anxiety and post-traumatic stress symptoms 35. Furthermore, a high proportion of the reported symptoms associated with COVID-19 (e.g., exercise intolerance, and respiratory or physical functional decline) have a potential effect on psychological health 13,14. Other published studies in patients with non-critical COVID-19 pneumonia have examined the impact of the disease on pulmonary function, health-related quality of life and perceived dyspnoea 36.

The cognitive dysfunction of patients with mild symptomatic COVID-19 has been also evaluated using the MoCA test and evidence of decline cognitive has been observed without establishing its pathogenesis 10. These previous findings are compatible with the cognitive sequelae observed in our study because the results from the MoCA test suggested a mild cognitive decline in the patients. Interestingly, although worse cognitive dysfunction was observed among older patients (≥ 65 years), anomalous results were also in younger patients. The present observations imply possible sequelae of COVID-19 from a neurological perspective, which supports the need for a multidisciplinary approach in the treatment of these patients 37.

Regarding the quality of life, Halpin and colleagues found a significant impact on quality of daily life in ICU survivors compared with patients with mild or moderate COVID-19 using the EQ-5D-5L scale 11. In contrast, other authors found similar results in the EQ-5D scale among patients with COVID-19 from different severity groups, and only slight differences in pain/discomfort were reported for patients with ICU stay 38. In agreement with this previous study, we showed a high percentage of "no problems" in four dimensions of the EQ-5D scale (i.e., mobility, self-care, usual activities, anxiety/depression) but a high percentage of “moderate pain or discomfort” was detected in pain/discomfort. Nevertheless, patients in our study perceived quality of life as good.

Major studies, such as the cohort study of Huang et al. with a sample of 1,733 patients, have reported sequelae after hospital discharge in patients with COVID-19 including sleeping difficulty, mood disorders, fatigue and muscular weakness 39. Muscular fatigue is a major limitation to exercise after pulmonary disease, probably before of muscular atrophy, prolonged periods confined to bed and hypoxia 40. It is remarkable that 76.0% of patients in our study reported some degree of fatigue after hospital discharge, with a progressive improvement during the multidisciplinary follow-up programme; in fact, about 50% of had no fatigue after completing the FACIT-F questionnaire at six-month phone interview follow-up.

Concerning dyspnoea, 64.4% of patients had some degree of mMRC dyspnoea and 37.5% required home oxygen therapy on hospital discharge. However, at six-month follow-up only 8.7% of patients needed oxygen. It is worth noting that 66 patients who required examination by a pneumologist showed, during the multidisciplinary follow-up circuit, normal spirometry findings, normal MIP for both sexes but a reduced MEP relative to the reference values 18, which suggests weakness in the strength of those muscles involved in expiration, such as the intercostals and abdominals. Accordingly, some publications argue that non-invasive support involves the risk of self-inflicted lung lesions by the patient, which negatively affects perceived symptoms 41,42. This means that some sequelae can be found independent of the respiratory therapy, although further studies are necessary to elucidate this matter. There are also studies comparing symptoms at 6 and 12 months after hospital discharge between patients who did not require oxygen therapy during admission and those who needed conventional high-flow oxygen therapy or mechanical ventilation 43. There are descriptions of abnormalities in pulmonary function on hospital discharge in these patients 44, which are compatible with interstitial lung disease and a high rate of persistent dyspnoea. Previously, ICU survivors had a dyspnoea score and pulmonary function similar to other patients, despite presenting worse results in the computerized tomography scan and lower performance in usual activities 45. Other studies found a decline of pulmonary function among critical patients, with a reduction in the distance of the 6-WMT and exercise-induced desaturation 46 as well as impairment in respiratory muscle strength 47. Respiratory function has been also studied for different patient profiles, including elderly patients, with special interest on frailty and fibromyalgia patients 48, 49. These studies found a correlation between hand grip strength (i.e., strength of peripheral muscles) and the strength of pulmonary muscles in comparison with healthy patients.

Rehabilitation plays a key role in the multidisciplinary follow-up of patients after hospital discharge 31,50. The concept of a hospital discharge after mechanical ventilation and ICU stay should be accompanied by early rehabilitation 51-53. In our study, the painful conditions described during rehabilitation visits were cervicobrachialgia, neuropathic pain, lumbar-sciatic pain, facet syndrome and multiple enthesopathies, with meralgia paresthetica as the most frequently described neuropathic pain. Regarding joint and muscular limitations, the main joint mobility problems were articular limitations in the shoulder and shoulder girdle; and the most frequent limitations on muscular mobility were in lower members, followed by upper members and general limitation. For distal neuropathy, we classified radial and cubital neuropathy in the upper members group and talipes equinovarus in the lower members group, finding 23.1% of patients with some type of distal neuropathy.

For evaluation of nutritional status, we considered BCM and BCMI 21 to detect the anomalous nutritional state of patients, and significant differences were observed. Overall, we found patients with a high BMI (only 6.7% of patients had a normal BMI) but a wide variation in BCMI; although the BCMI means for both men and women were lower than the reference values 21, which indicates undernourishment despite weight loss during hospitalisation.

The main point of interest in this observational study is the multidisciplinary nature of the sequelae suffered by patients who were admitted to the ICU for severe COVID-19 pneumonia at our hospital. In this respect, the Hospital Universitario Virgen de la Victoria pioneered the treatment of patients with severe hypoxia using high-flow nasal cannula therapy in the ICU, unlike other centres where these patients remained on general wards. It is worth noting that, even though orotracheal intubation was necessary for 53.8% of the patients, the sensation of dyspnoea and perceived fatigue during follow-up reached 64.2% and 76.0% respectively, and the general sequelae described after multidisciplinary evaluation were considerable with about 78% of patients requiring rehabilitation.

The main limitations of this study are the small sample size and the fact that not all the patients were examined and evaluated by all the specialties who were included in the multidisciplinary programme, which could imply bias in the interpretation of the results; for instance, certain tests, such as spirometry, were only conducted in patients who had a bad respiratory prognosis on hospital discharge. It would have been beneficial the inclusion of patients during the post-COVID follow-up without requiring ICU admission in order to report on a broader and more complete range of sequelae after home discharge patients. A long-term follow-up and an increase in the sample size are necessary to better characterise the consequences of COVID-19.

## Conclusions

We conclude that a post-ICU multidisciplinary approach in patients with severe COVID-19 pneumonia is fundamental because of the wide range of sequelae. The role of early rehabilitation is key to minimise the disabling effect of these sequelae to optimise the extent of independence and maximise the reintegration in society of these patients.

## Figures and Tables

**Table 1:** Baseline sociodemographic and clinical characteristics of the patients

VARIABLE		RESULT
**Sex** [*n* (%)]	*n* Man Woman	104 81 (77.9) 23 (22.1)
**Age (> 60 years)** [*n* (%)]		54 (51.9)
**Age** [mean (SD)]		56.69 (13.76)
**BMI (kg/m^2^)** [*n* (%)]	Normal (18.5-24.9) Overweight (25-29.9) Obese 1 (30-34.9) Obese 2 (35-39.9) Morbid obese (>40)	7 (6.7) 31 (29.8) 32 (30.8) 18 (17.3) 16 (15.4)
**Smoking** [*n* (%)]	Smoker Ex-smoker Non-smoker	4 (3.8) 46 (44.2) 54 (51.9)
**Comorbidity** [*n* (%)]	High blood pressure Diabetes Dyslipidaemia	50 (48.1) 29 (27.9) 32 (30.8)
**Respiratory history** [*n* (%)]	Total COPD Asthma OSA	23 (22.2) 3 (2.9) 9 (8.7) 11 (10.6)
**Cardiological history** [*n* (%)]	Total Ischaemic heart disease Arrhythmias Valvular heart disease Other	11 (10.6) 5 (4.8) 3 (2.9) 2 (1.9) 1 (1.0)
**Orthopaedic history** [*n* (%)]	Total Prior fractures Arthritis Myalgia Osteoarthritis	8 (7.7) 4 (3.8) 1 (1.0) 1 (1.0) 2 (1.9)
**Kidney disease** [*n* (%)]		2 (1.9)
**Duration ICU stay (days)** [mean (SD)]		21.87 (19.64)
**Duration hospitalization (days)** [mean (SD)]		47.34 (49.56)
**COVID-19 treatment(s)**	High-flow nasal cannula [*n* (%)] Invasive mechanical ventilation [*n* (%)] Tracheotomy [*n* (%)] ECMO [*n* (%)] Mechanical ventilation (days) [mean (SD)] Delay intubation (days) [mean (SD)] Prone positioning (days) [mean (SD)]	48 (46.2) 56 (53.8) 34 (32.7) 1 (0.96) 27.14 (21.58) 1.33 (2.16) 3.70 (2.12)
**Pharmacological treatment(s)**	Hydroxychloroquine [*n* (%)] Tocilizumab [*n* (%)] Interferon beta [*n* (%)] Lopinavir-ritonavir [*n* (%)] Muscle relaxant (days) [mean (SD)]	22 (21.2) 61 (58.7) 18 (17.3) 20 (19.2) 7.70 (6.72)

Abbreviations: BMI: body mass index; ECMO: extracorporeal membrane oxygenation; COPD: chronic obstructive pulmonary disease; OSA: obstructive sleep apnoea.

**Table 2:** Sequelae detected in patients who suffered from severe COVID-19 pneumonia after hospital discharge

VARIABLE		RESULT
**Weight loss post-ICU (kg) [**mean (SD)**]**		10.76 (7.86)
**BCM (kg) in men [**mean (SD)**]**		28.43 (6.71)
**BCM (kg) in women [**mean (SD)**]**		21.30 (11.97)
**BCMI (kg) in men [**mean (SD)**]**		9.59 (2.54)
**BCMI (kg) in women [**mean (SD)**]**		9.54 (6.51)
**ENT [***n* (%)**]**	Total Aphonia Glottic/aural granuloma Tracheal stenosis Hearing loss Tracheostomy fistula	23 (22.11) 14 (13.46) 3 (2.88) 3 (2.88) 2 (1.92) 1 (0.96)
**Psychological/psychiatric disorders [***n* (%)**]**	Normal experiential reaction Anxiety Depression Anxiety-depressive disorder Adjustment disorder	11 (10.58) 6 (5.77) 3 (2.88) 8 (7.69) 12 (11.54)
**Insomnia [***n* (%)**]**		27 (25.96)
**Benzodiazepine consumption [***n* (%)**]**		30 (28.84)
**Antidepressant consumption [***n* (%)**]**		28 (26.92)
**mMRC dyspnoea [***n* (%)**]**	Grade 0 Grade 1 Grade 2 Grade 3	37 (35.58) 33 (31.73) 25 (24.04) 9 (8.65)
**Home oxygen therapy [***n* (%)**]**	On hospital discharge At 6-month follow-up	39 (37.50) 9 (8.65)
**Joint mobility [***n* (%)**]**	Normal Shoulder limitations Shoulder girdle limitation Wrist limitations Hip limitations Lower limb limitations	66 (63.46) 12 (11.54) 3 (2.88) 2 (1.92) 1 (0.96) 1 (0.96)
**Muscular mobility [***n* (%)**]**	Normal Gait abnormalities General limitation Shoulder girdle limitation Upper limb limitations Lower limb limitations	51 (49.04) 1 (0.96) 7 (6.73) 3 (2.88) 6 (5.77) 17 (16.35)
**Painful conditions [***n* (%)**]**	Total Cervicobrachialgia Neuropathic Lumbar-sciatic Facet syndrome Multiple enthesopathies Meralgia paresthetica	28 (26.92) 1 (0.96) 14 (13.46) 5 (4,81) 1 (0.96) 6 (5.77) 8 (7.69)
**Distal neuropathy [***n* (%)**]**	Total Upper limb Lower limb Upper & lower limb	24 (23.08) 9 (8.65) 16 (15.38) 3 (2.88)
**Brachial plexopathy [***n* (%)**]**		6 (5.77)
**Talipes equinovarus [***n* (%)**]**	Unilateral Bilateral	5 (4,81) 3 (2.88)
**Aid [***n* (%)**]**	Without aid Walking stick/frame Wheelchair	85 (81.73) 16 (15.38) 3 (2.88)
**Fatigue/tiredness [***n* (%)**]**	Total Mild effort Moderate effort Great effort	79 (75.96) 10 (9.62) 50 (48.08) 19 (18.27)
**MoCA [**mean (SD)**]**		21.35 (9.93)

Abbreviations: BCM: body cell mass; BCMI: body cell mass index; ENT: ear, nose and throat - otorhinolaryngology; mMRC: modified medical research council dyspnea scale; MoCA: Montreal cognitive assessment.

**Table 3:** Rehabilitation functional testing with the short physical performance battery, the six-minute walk test and grip strength with a hand dynamometer

VARIABLE		RESULT
**SPPB score [***n* (%)**]**	*n* Normal Frail	48 35 (72.92) 13 (27.08)
**6-MWT [***n* (%)**]**	*n* Normal distance Distance < 350 m 0 stops 1 stop 2 or more stops Oxygen	46 29 (63.04) 17 (36.96) 40 (86.96) 4 (8.69) 2 (4.35) 3 (6.52)
**Grip strength (kg) [**mean (SD)**]**	*n* Right hand Left hand	81 25.06 (10.63) 22.35 (11.26)

Abbreviations: SPPB: short physical performance battery; 6-MWT: six-minute walk test.

**Table 4:** Respiratory functional testing with spirometry, maximal inspiratory pressure and maximal expiratory pressure

VARIABLE		RESULT
**Spirometry** [mean (SD)]	*n* FVC (%) FEV1 (%) FEV1/FVC ratio (%)	66 87.36 (19.87) 81.00 (22.29) 75.06 (12.19)
**MIP and MEP in men** [mean (SD)]	*n* MIP MEP	66 81.64 (29.80) 95.60 (41.64)
**MIP and MEP in women** [mean (SD)]	*n* MIP MEP	17 68.80 (43.96) 70.20 (22.24))

Abbreviations: FEV1: forced expiratory volume in first second of forced breath; FVC: forced vital capacity; MEP: maximal expiratory pressure; MIP: maximal inspiratory pressure.

**Table 5:** The European quality of life-5 dimensions questionnaire

VARIABLE		RESULT
**EQ-5D: perceived quality of life (points)** [mean (SD)]		60.62 (20.15)
**Mobility** [*n* (%)]	No problems Moderate problems Severe problems	35 (62.50) 19 (33.93) 1 (1.78)
**Self-care** [*n* (%)]	No problems Moderate problems Severe problems	38 (67.86) 15 (26.79) 2 (3.57)
**Usual activities** [*n* (%)]	No problems Moderate problems Severe problems	28 (50.00) 26 (46.43) 1 (1.78)
**Pain/discomfort** [*n* (%)]	No problems Moderate problems Severe problems	25 (44.64) 28 (50.00) 2 (3.57)
**Anxiety/depression** [*n* (%)]	No problems Moderate problems Severe problems	36 (64.29) 15 (26.79) 4 (7.14)

Abbreviations: EQ-5D: European quality of life-5 dimensions questionnaire.

## Abbreviations

6-MWT: six-minute walk test
ARDS: acute respiratory distress syndrome
BCM: body cell mass
BCMI: body cell mass index
BMI: body mass index
COPD: chronic obstructive pulmonary disease
COVID-19: coronavirus disease 2019
ECMO: extracorporeal membrane oxygenation
ENT: ear, nose and throat - otorhinolaryngology
EQ-5D: European quality of life-5 dimensions questionnaire
FACIT-F: functional assessment of chronic illness therapy-fatigue scale
FEV1: forced expiratory volume in first second of forced breath
FVC: forced vital capacity
ICU: intensive care unit
MEP: maximal expiratory pressure
MIP: maximal inspiratory pressure
mMRC: modified medical research council dyspnea scale
MoCA: Montreal cognitive assessment
OSA: obstructive sleep apnoea
PaO2: partial pressure of oxygen in arterial blood
PICS: post intensive care syndrome
SD: standard deviation
SPPB: short physical performance battery

## References

[1] Pipolo C, Bulfamante AM, Schillaci A, Banchetti J, Castellani L, Saibene AM (2021). Through The Back Door: Expiratory Accumulation of SARS-Cov-2 in the Olfactory Mucosa as Mechanism for CNS Penetration. Int J Med Sci.

[2] Li J (2022). Rehabilitation management of patients with COVID-19: lessons learned from the first experience in China. Eur J Phys Rehabil Med [Internet]. 2020 Jul [cited.

[4] Smith S, Rahman O (2022). Post Intensive Care Syndrome. In: StatPearls [Internet]. Treasure Island (FL): StatPearls Publishing; 2022 [cited.

[5] Theeranut A, Methakanjanasak N, Surit P, Srina J, Sirivongs D, Adisuksodsai D (2021). Can a multidisciplinary approach slow renal progression in CKD patients?. Int J Med Sci.

[6] Rawal G, Yadav S, Kumar R (2017). Post-intensive care syndrome: An overview. J Transl Intern Med.

[7] Needham DM, Davidson J, Cohen H, Hopkins RO, Weinert C, Wunsch H (2012). Improving long-term outcomes after discharge from intensive care unit: Report from a stakeholdersʼ conference*. Crit Care Med.

[8] Chan K, Zheng J, Mok Y, Li Y, Liu YN, Chu C (2003). SARS: prognosis, outcome and sequelae. Respirology.

[9] Wang Y, Chen X, Wang F, Geng J, Liu B, Han F (2021). Value of anal swabs for SARS-COV-2 detection: a literature review. Int J Med Sci.

[10] Del Brutto OH, Wu S, Mera RM, Costa AF, Recalde BY, Issa NP (2021). Cognitive decline among individuals with history of mild symptomatic SARS-CoV-2 infection: A longitudinal prospective study nested to a population cohort. Eur J Neurol.

[11] Halpin SJ, McIvor C, Whyatt G, Adams A, Harvey O, McLean L (2021). Postdischarge symptoms and rehabilitation needs in survivors of COVID-19 infection: A cross-sectional evaluation. J Med Virol.

[12] Neufeld KJ, Leoutsakos JMS, Yan H, Lin S, Zabinski JS, Dinglas VD (2020). Fatigue Symptoms During the First Year Following ARDS. Chest.

[13] Bellan M, Soddu D, Balbo PE, Baricich A, Zeppegno P, Avanzi GC (2021). Respiratory and Psychophysical Sequelae Among Patients With COVID-19 Four Months After Hospital Discharge. JAMA Netw Open.

[14] Arnold DT, Hamilton FW, Milne A, Morley AJ, Viner J, Attwood M (2021). Patient outcomes after hospitalisation with COVID-19 and implications for follow-up: results from a prospective UK cohort. Thorax.

[15] Boldrini P, Kiekens C, Bargellesi S, Brianti R, Galeri S, Lucca L (2022). First impact of COVID-19 on services and their preparation. “Instant paper from the field” on rehabilitation answers to the COVID-19 emergency. Eur J Phys Rehabil Med [Internet]. 2020 Jul [cited.

[16] Negrini S, Ferriero G, Kiekens C, Boldrini P (2022). Facing in real time the challenges of the COVID-19 epidemic for rehabilitation. Eur J Phys Rehabil Med [Internet]. 2020 Jul [cited.

[17] Álvarez Gutiérrez FJ, Barchilón Cohen V, Casas Maldonado F, Compán Bueno MV, Entrenas Costa LM, Fernández Guerra J (2009). Documento de Consenso sobre la espirometría en Andalucía. SEMERGEN - Med Fam.

[18] Mora-Romero U de J, Gochicoa-Rangel L, Guerrero-Zúñiga, Selene (2014). Presiones inspiratoria y espiratoria máximas: Recomendaciones y procedimiento. Neumol Cir Torax.

[19] Cabasés JM (2015). El EQ-5D como medida de resultados en salud. Gac Sanit.

[20] Barbosa-Silva MCG Subjective and objective nutritional assessment methods: what do they really assess?: Curr Opin Clin Nutr Metab Care. 2008 May;11(3):248-54.

[21] Talluri A, Liedtke R, Mohamed EI, Maiolo C, Martinoli R, De Lorenzo A (2003). The application of body cell mass index for studying muscle mass changes in health and disease conditions. Acta Diabetol.

[22] Guralnik JM, Simonsick EM, Ferrucci L, Glynn RJ, Berkman LF, Blazer DG (1994). A Short Physical Performance Battery Assessing Lower Extremity Function: Association With Self-Reported Disability and Prediction of Mortality and Nursing Home Admission. J Gerontol.

[23] Luna Heredia E, Martin Pena G, Ruiz Galiana J (2004). Valores normales y límites de la normalidad de la fuerza de la mano determinados con dinamometría. Nutr Hosp.

[24] Rantanen T (1999). Midlife Hand Grip Strength as a Predictor of Old Age Disability. JAMA.

[26] Lai JS, Cook K, Stone A, Beaumont J, Cella D (2009). Classical test theory and item response theory/Rasch model to assess differences between patient-reported fatigue using 7-day and 4-week recall periods. J Clin Epidemiol.

[27] Chang CH, Cella D, Clarke S, Heinemann AW, Von Roenn JH, Harvey R (2003). Should symptoms be scaled for intensity, frequency, or both?. Palliat Support Care.

[28] Cid-Ruzafa J, Damian-Moreno J (1997). Valoración de la discapacidad física: el indice de Barthel. Rev Esp Salud Publica.

[29] Nasreddine ZS, Phillips NA, BÃdirian V, Charbonneau S, Whitehead V, Collin I (2005). The Montreal Cognitive Assessment, MoCA: A Brief Screening Tool For Mild Cognitive Impairment: MOCA: A BRIEF SCREENING TOOL FOR MCI. J Am Geriatr Soc.

[30] Pedraza OL, Sanchez E, Plata S (2014). Puntuaciones del MoCA y el MMSE en pacientes con deterioro cognitivo leve y demencia en una clínica de memoria en Bogotá. Acta Neurol Colomb.

[31] Puchner B, Sahanic S, Kirchmair R, Pizzini A, Sonnweber B, Wöll E (2022). Beneficial effects of multi-disciplinary rehabilitation in postacute COVID-19: an observational cohort study. Eur J Phys Rehabil Med [Internet]. 2021 May [cited.

[32] Simpson R, Robinson L (2020). Rehabilitation After Critical Illness in People With COVID-19 Infection. Am J Phys Med Rehabil.

[33] Hui DS, Wong KT, Ko FW, Tam LS, Chan DP, Woo J (2005). The 1-Year Impact of Severe Acute Respiratory Syndrome on Pulmonary Function, Exercise Capacity, and Quality of Life in a Cohort of Survivors. Chest.

[34] Ramani C, Davis EM, Kim JS, Provencio JJ, Enfield KB, Kadl A (2021). Post-ICU COVID-19 Outcomes. Chest.

[35] Weidman K, LaFond E, Hoffman KL, Goyal P, Parkhurst CN, Derry-Vick H (2021). Post-ICU Syndrome in a Cohort of COVID-19 Survivors in New York City. Ann Am Thorac Soc.

[36] van der Sar - van der Brugge S, Talman S, Boonman - de Winter L, de Mol M, Hoefman E, van Etten RW (2021). Pulmonary function and health-related quality of life after COVID-19 pneumonia. Respir Med.

[37] Rota V, Redolfi A, Monteleone S, Arienti C, Falso M (2022). Can COVID-19 result in cognitive dysfunctions? The need for a multidisciplinary approach in rehabilitation for post-COVID-19 people. Eur J Phys Rehabil Med [Internet]. 2022 Mar [cited.

[38] Garrigues E, Janvier P, Kherabi Y, Le Bot A, Hamon A, Gouze H (2020). Post-discharge persistent symptoms and health-related quality of life after hospitalization for COVID-19. J Infect.

[39] Huang C, Huang L, Wang Y, Li X, Ren L, Gu X (2021). 6-month consequences of COVID-19 in patients discharged from hospital: a cohort study. The Lancet.

[40] Carda S, Invernizzi M, Bavikatte G, Bensmaïl D, Bianchi F, Deltombe T (2022). COVID-19 pandemic. What should Physical and Rehabilitation Medicine specialists do? A clinician's perspective. Eur J Phys Rehabil Med [Internet]. 2020 Sep [cited.

[41] Grieco DL, Maggiore SM, Roca O, Spinelli E, Patel BK, Thille AW (2021). Non-invasive ventilatory support and high-flow nasal oxygen as first-line treatment of acute hypoxemic respiratory failure and ARDS. Intensive Care Med.

[42] González-Castro A, Fajardo Campoverde A, Roncalli A (2021). Cánulas nasales de alto flujo en la neumonía por COVID-19. Med Clínica.

[43] Huang C, Huang L, Wang Y, Li X, Ren L, Gu X (2021). 6-month consequences of COVID-19 in patients discharged from hospital: a cohort study. The Lancet.

[44] Sonnweber T, Sahanic S, Pizzini A, Luger A, Schwabl C, Sonnweber B (2021). Cardiopulmonary recovery after COVID-19: an observational prospective multicentre trial. Eur Respir J.

[45] Lerum TV, Aaløkken TM, Brønstad E, Aarli B, Ikdahl E, Lund KMA (2021). Dyspnoea, lung function and CT findings 3 months after hospital admission for COVID-19. Eur Respir J.

[46] Guler SA, Ebner L, Aubry-Beigelman C, Bridevaux PO, Brutsche M, Clarenbach C (2021). Pulmonary function and radiological features 4 months after COVID-19: first results from the national prospective observational Swiss COVID-19 lung study. Eur Respir J.

[47] Huang Y, Tan C, Wu J, Chen M, Wang Z, Luo L (2020). Impact of coronavirus disease 2019 on pulmonary function in early convalescence phase. Respir Res.

[48] Parentoni AN, Mendonça VA, Dos Santos KD, Sá LF, Ferreira FO, Gomes Pereira DA (2015). Gait speed as a predictor of respiratory muscle function, strength, and frailty syndrome in community-dwelling elderly people. J Frailty Aging.

[49] Şahin G, Ulubaş B, Çalkoǧlu M, Erdoǧan C Handgrip Strength, Pulmonary Function Tests, and Pulmonary Muscle Strength in Fibromyalgia Syndrome: Is There Any Relationship?: South Med J. 2004 Jan;97(1):25-9.

[50] Curci C, Negrini F, Ferrillo M, Bergonzi R, Bonacci E, Camozzi DM (2022). Functional outcome after inpatient rehabilitation in postintensive care unit COVID-19 patients: findings and clinical implications from a real-practice retrospective study. Eur J Phys Rehabil Med [Internet]. 2021 Jul [cited.

[51] Stam H, Stucki G, Bickenbach J (2020). Covid-19 and Post Intensive Care Syndrome: A Call for Action. J Rehabil Med.

[52] Khan F, Amatya B, Gosney J, Rathore FA, Burkle FM (2015). Medical Rehabilitation in Natural Disasters: A Review. Arch Phys Med Rehabil.

[53] Expósito Tirado JA, Rodríguez-Piñero Durán M, Echevarría Ruiz de Vargas C (2020). Rehabilitación médica y COVID-19: impacto actual y retos futuros en los servicios de rehabilitación. Rehabilitación.

